# Transoral Laser Microsurgery versus Robot-Assisted Surgery for Squamous Cell Carcinoma of the Tongue Base (Oncological and Functional Results)—A Retrospective GETTEC Multicenter Study

**DOI:** 10.3390/jcm12134210

**Published:** 2023-06-22

**Authors:** Ioana Brudasca, Pierre Philouze, Sylvain Morinière, Benjamin Lallemant, Sébastien Vergez, Olivier Malard, Pierre-Eric Roux, Noémie Rossello, Caroline Payen, Philippe Céruse

**Affiliations:** 1 Service d’ORL et de Chirurgie Cervico-Faciale, Centre Hospitalier Croix Rousse, Hospices Civils de Lyon, 69004 Lyon, Francephilippe.ceruse@chu-lyon.fr (P.C.); 2 Service d’ORL et Chirurgie Cervico-Faciale, Centre Hospitalier Régional Universitaire Bretonneau, 37000 Tours, France; 3 Service d’ORL et Chirurgie Cervico-Faciale, Centre Hospitalier Universitaire de Nîmes, 30900 Nîmes, France; 4 Service d’ORL et Chirurgie Cervico-Faciale, Oncopole, Institut Universitaire du Cancer de Toulouse, 31059 Toulouse, France; 5 Service d’ORL et Chirurgie Cervico-Faciale, Centre Hospitalier Universitaire de Nantes, 44093 Nantes, France; 6 Service d’ORL et Chirurgie Cervico-Faciale, Centre Léon Bérard, 69008 Lyon, France

**Keywords:** oropharynx squamous cell carcinoma, base of tongue, TORS, TLM

## Abstract

The base of the tongue (BOT) is the second most common site for squamous cell carcinoma (SCC) in the oropharynx. There are currently no clear guidelines for the management of BOT SCC. Our main objective was to compare the oncological outcomes of two minimally invasive approaches, transoral laser microsurgery (TLM) and transoral robot-assisted surgery (TORS). This was a retrospective French GETTEC (Groupe d’Études des Tumeurs de la Tête et du Cou) multicenter study of patients with BOT SCC removed surgically either by TLM or TORS between 2005 and 2021. The study group included 16 patients treated by TLM and 38 by TORS, with median follow-up times of 14.4 and 37.2 months, respectively. The overall survival (OS) rates at 2 and 3 years were 67% in the TLM group and 90% at 2 years and 86% at 3 years in the TORS group (*p* = 0.42, *p* = 0.20). There was no significant difference in recurrence-free survival (RFS) between the two techniques after 2 and 3 years. The tumors removed by TORS were significantly larger. Operative times were significantly shorter in the TLM group. There were no differences in feeding resumption; none of the patients in the TLM group required a tracheotomy. Postoperative hemorrhagic complication rates were similar in the two groups (12% for TLM and 13% for TORS). Both TORS and TLM showed encouraging oncological, functional, and safety results in BOT SCC even in recurrence or second primary cancer patients, without a technique being found superior in terms of OS or RFS. Tumors removed by TORS were larger without an increase in postoperative bleeding, extending the possibilities of transoral treatment.

## 1. Introduction

Tumors of the oropharynx are very heterogeneous, differing in accessibility and histological nature. More than 90% are squamous cell carcinomas (SCCs), with an increasing prevalence of HPV-related malignancies affecting younger patients with fewer comorbidities [1,2]. These patients with virus-induced cancer have better prognoses and higher survival rates than those with tobacco- or alcohol-related tumors. The functional consequences of cancer treatment and post-treatment quality of life are therefore particularly important to consider [3].

The most common site for oropharynx squamous cell carcinomas (OPSCCs) after the tonsils is the base of the tongue (BOT) [4]. There are no clear guidelines for the management of these tumors; medical treatment (chemo- or radiotherapy) and upfront surgery are both possible. Surgical resection must ensure negative margins and preserve the key role of the tongue in swallowing and speech. Endoscopic mini-invasive surgery is a good treatment option for OPSCC [5,6], even if some tumors can be difficult to access and expose.

Transoral treatment of OPSCC was first described by Huet in 1951 [7] and has since been widely adopted. Transoral approaches, which nowadays include transoral laser microsurgery (TLM) and transoral robotic surgery (TORS), reduce operating times and scarring and improve functional outcomes compared with open surgery. 

Transoral laser microsurgery (TLM) is a validated treatment option for BOT SCC, including advanced tumors [8,9], while TORS was approved by the FDA in 2009 for the treatment of T1-T2 OPSCC [10]. This has led to numerous comparative studies of the functional and oncologic outcomes of TORS with those of open or other endoscopic approaches [11,12,13,14,15,16,17]. Most of these studies consider all OPSCC tumor sites, with a minority of BOT SCCs included [12,15] and often without stratification by anatomical location. While comparing TLM and TORS specifically for BOT SCC seems important, this is to our knowledge the first study on the subject.

Our main objective was to compare the oncological outcomes of minimally invasive transoral surgery by TLM or TORS in patients with SCC of the BOT. Functional outcomes, postoperative complications (hemorrhage, tracheostomy, feeding resumption), and operating times with the two techniques were also compared.

## 2. Methods

### 2.1. Type of Study

This was a retrospective French GETTEC multicenter study of adult patients with BOT SCC treated by TLM or TORS between 2005 and 2021. Data were collected from the medical records of six French tertiary referral hospitals. 

### 2.2. Inclusion and Exclusion Criteria

Patients were included if they had isolated BOT SCC, or BOT SCC with limited extension to the vallecula or tonsils; T1 and T2 stage tumors were included if they did not meet the exclusion criteria. 

The exclusion criteria were as follows: age < 18 years, multi-metastatic evolution of the pathology before surgery, synchronous neoplastic lesions of the upper aerodigestive tract (UAT), BOT SCC with extension beyond the glossotonsillar sulcus or the vallecula (involvement of the lateral or posterior pharyngeal wall, the veil, the intermaxillary commissure, the movable tongue, the floor of the mouth or the epiglottis), non-SCC tumor, patients nonexposable for transoral approach.

### 2.3. Surgical Technique

TLM was used in one treatment center, and TORS was used in the five others. All TLM procedures were performed by trained surgeons with a super pulse CO_2_ laser (continuous 5-watt setting). All TORS procedures were performed with the DaVinci system, by trained operators. The need for lymph node dissection was evaluated based on the location of the tumor and patients' history of lymph node dissection.

### 2.4. Data Collected

The data collected from the patients’ medical records included demographic (age, sex, care center, deceased or not, and cause of death) and clinical variables (comorbidities, treatments, ASA Physical Status classification [18], history of UAT cancer and prior UAT cancer treatment, tumor location and management).

The information gathered on tumor management included the date of histologically confirmed diagnosis of SCC, date and type of transoral surgery, precise tumor location and cTNM staging, whether or not cervical lymph node dissection were performed, whether or not lingual artery ligature was performed, transoral operative time, and adjuvant treatments. The transoral operating times collected in the anesthesiology reports included robot or laser set-up time but excluded cervical operating time. Information on local, regional, or metastatic recurrences related to their delay and management was also recorded.

The pathological data collected were the HPV status of the tumor (determined by p16 immunochemistry), pTNM staging, and margin status. Margin status was considered negative if >5 mm and close margin if <5 mm [15].

Functional outcomes were assessed in terms of the length of hospital stay, postoperative respiratory or bleeding complications requiring revision surgery or not, tracheostomy requirement and weaning time, nasogastric enteral nutrition requirement and weaning time, or secondary gastrostomy. 

### 2.5. Main Outcome Variables

The main outcome variables were the overall survival (OS) and local (LRFS), regional (RRFS), and metastatic recurrence-free survival (MRFS) status at 2 and 3 years.

### 2.6. Secondary Outcome Variables

We compared the size (bigger axis) and the quality of the resection margins, the respiratory outcomes (tracheostomy requirement), the resumption of feeding (nasogastric feeding requirement and duration, gastrostomy requirement), transoral operative and hospitalization times, and postoperative respiratory or bleeding complication rates. 

### 2.7. Statistical Analysis

Statistical analyses were performed using Excel and R (version 4.0.3, R Development Core Team, Vienna, Austria, 2021). Quantitative variables were compared using chi-square tests or Fischer’s exact test. Categorical variables were compared using Student’s *t*-test or Welch's test when variances were unequal. Survival analysis was performed using log-rank tests. Results were considered statistically significant at *p* < 0.05 and were validated by members of the study center’s biostatistics department. 

### 2.8. Ethics

The study was conducted in accordance with the Declaration of Helsinki and approved by the local ethics committee on 11 February 2022 (approval No. 22_619). The data collected were strictly anonymous and outside the scope of French data protection laws. Patients provided informed consent at the time of treatment for their data to be used in future scientific studies.

## 3. Results

Fifty-four patients were included, 16 treated with TLM and 38 with TORS. Preliminary analysis showed no statistically significant differences in terms of preoperative characteristics between the two groups (Table 1).

A majority of patients were treated for a recurrence or second primary UAT SCC—63% (10/16) in the TLM group (1 recurrence and 9 second primary tumors) and 42% (16/38) in the TORS group (2 recurrences and 14 second primary tumors). Recurrence or second primary tumor occurred in irradiated areas in 90% (9/10) of patients in the TLM group and in 60% (12/16) of patients in the TORS group. 

Tumor stages did not differ significantly overall between the two groups. HPV status was not documented for all patients; 53% of SCCs were P16-positive in the TLM group (8/15), and 45% of SCCs were P16-positive in the TORS group (13/29).

Positive surgical margins were observed for 25% of patients in the TLM group (4/16) and 15.7% of patients in the TORS group (6/38). Lymph node dissection was performed in 43% of patients in the TLM group (7/16) and 68% of patients in the TORS group (26/38). Lingual artery ligature was performed in 12% of patients in the TLM group (2/16) and 27% of patients in the TORS group (10/34). 

Patients treated by TORS were followed up for longer on average than those who underwent TLM (mean follow-up time, 37.2 months versus 14.4 months). 

### 3.1. Oncological Outcomes

**Figure 1:**
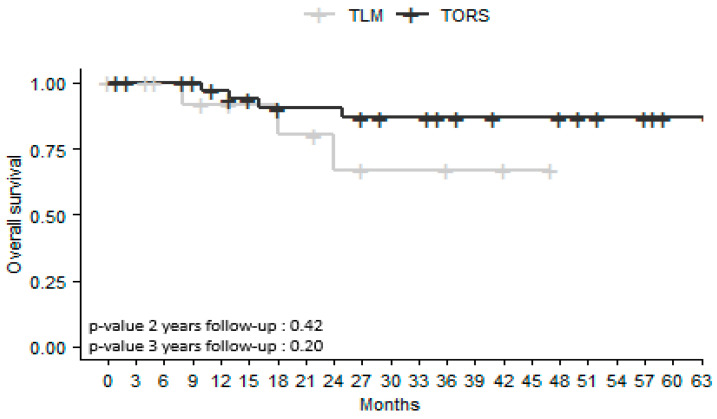
Kaplan–Meier survival curves for overall survival in patients with squamous cell carcinoma of the base of the tongue treated by transoral laser microsurgery (TLM) or transoral robotic surgery (TORS). Number of censored cases at 2 years: TORS *n* = 8; TLM *n* = 6. At 3 years: TORS *n* = 12; TLM *n* = 8.

OS rates did not differ significantly between the TORS and TLM groups, either 2 years (*p* = 0.42) or 3 years after surgery (*p* = 0.20). The survival rate was 67% at 2 years and at 3 years in the TLM group and 90% at 2 years and 86% at 3 years in the TORS group (Figure 1). 

**Figure 2:**
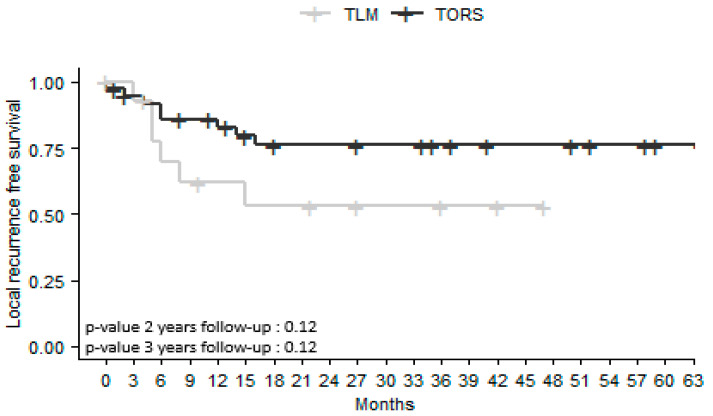
Kaplan–Meier survival curves for local recurrence-free survival in patients with squamous cell carcinoma of the base of the tongue treated by transoral laser microsurgery (TLM) or transoral robotic surgery (TORS). Number of censored cases at 2 years: TORS *n* = 7; TLM *n* = 4. At 3 years: TORS *n* = 10; TLM *n* = 6.

Local recurrence-free survival (LRFS) was higher in the TORS group both 2 and 3 years after surgery (76% versus 53% in the TLM group; *p* = 0.12). Recurrences occurred less than 12 months after initial surgery for 4/5 patients in the TLM group and 7/8 in the TORS group; 1/5 occurred less than 24 months after initial surgery in the TLM group; 1/8 occurred 2 years after the initial surgery in the TORS group (Figure 2).

**Figure 3:**
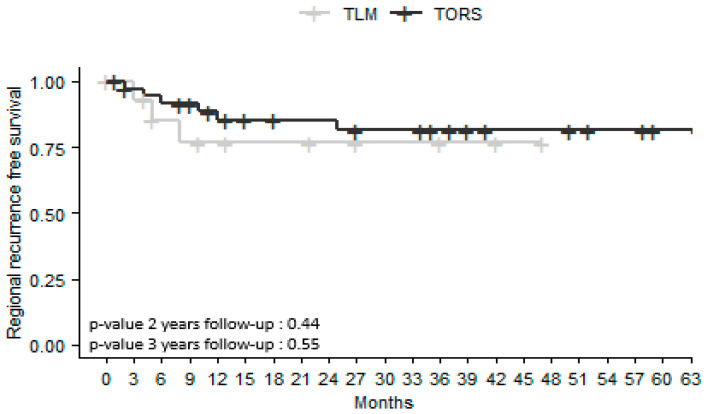
Kaplan–Meier survival curves for cervical lymph node recurrence-free survival in patients with squamous cell carcinoma of the base of the tongue treated by transoral laser microsurgery (TLM) or transoral robotic surgery (TORS). Number of censored cases at 2 years: TORS *n* = 8; TLM *n* = 6. At 3 years: TORS *n* = 11; TLM *n* = 8.

**Figure 4:**
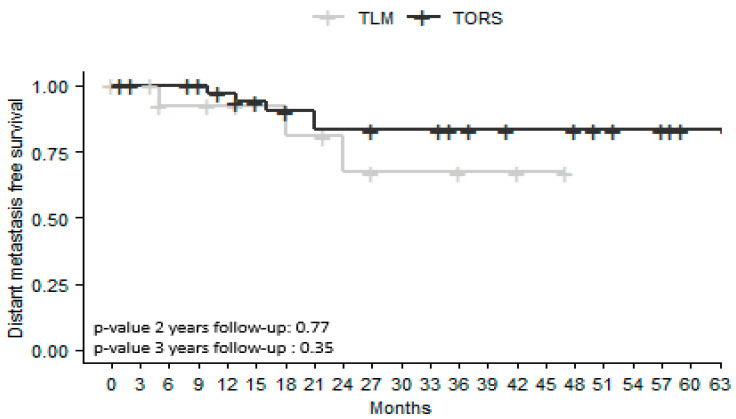
Kaplan–Meier survival curves for metastasis-free survival in patients with squamous cell carcinoma of the base of the tongue treated by transoral laser microsurgery (TLM) or transoral robotic surgery (TORS). Number of censored cases at 2 years: TORS *n* = 8; TLM *n* = 6. At 3 years: TORS *n* = 11; TLM *n* = 8.

There were no significant differences between the two groups in the rates of RRFS and MRFS at 2 and 3 years (Figure 3 and Figure 4). Regional recurrence-free survival (RRFS) was higher in the TORS group both 2 and 3 years after surgery (76% versus 85% in the TLM group, *p* = 0.44 at 2-year follow-up; and 76% versus 81% in the TLM group, *p* = 0.55 at 3-year follow-up). Metastatic recurrence-free survival (MRFS) was higher in the TORS group both 2 and 3 years after surgery (67% versus 83% in the TLM group, *p* = 0.77 at 2-year follow-up; 67% versus 83% in the TLM group, *p* = 0.35 at 3-year follow-up).

Histopathological characteristics are summarized in Table 2. There were no significant differences between the two groups in the proportions of patients with close tumor margins (25% (4/16) in the TLM group versus 15% (6/38) in the TORS group; *p* = 0.45). The tumors removed by TORS were significantly larger than those removed by TLM (longest axis 31 (±10) mm in the TLM group versus 39 (±14) mm in the TORS group; *p* = 0.04). There was no difference in pT stage (*p* = 0.35).

There was no statistically significant difference in the proportion of patients receiving adjuvant radiotherapy (31% (5/16) after TLM versus 63% (24/38) after TORS; *p* = 0.06) or chemo-radiotherapy (6% (1/16) after TLM versus 24% (9/38) after TORS; *p* = 0.25).

### 3.2. Functional Results

Functional results are summarized in Table 3. Operating times (laser installation/docking included) were significantly shorter in the TLM group (median [interquartile range], 59 [24–96] min versus 75 [55–158] min in the TORS group; *p* = 0.03). Note that transoral operating times did not decrease during the study period even if laser installation and docking times did. Hospitalization times did not differ significantly, with median lengths of hospital stay of 5 [2.5–9] days in the TLM group and 8 [7–11] days in the TORS group (*p* = 0.2). 

Patients treated by TORS had a tracheotomy in 18% of cases (7/38). None of the TLM patients had a tracheotomy (*p* = 0.05). The mean tracheotomy duration was 10 (± 6) days. None of the patients had postoperative upper airway obstruction requiring secondary tracheotomy.

There were no significant differences between the two groups in the need for or duration of nasogastric enteral feeding: 50% of TLM patients (8/16) with a median duration of 4 [2.8–9.8] days versus 86% of TORS patients (26/38) with a median duration of 7 [5.8–34.2] days (*p* = 0.33; *p* = 0.61). Two patients in the TLM group (12%) and five patients in the TORS group (13%) required long-term enteral feeding via a gastrostomy (*p* = 1).

The rates of postoperative bleeding were similar in the two groups: 12% in the TLM group (2/16 patients) and 13% in the TORS group (5/38 patients; *p* = 1). Bleeding complications required revision surgery in 6/8 cases, on average 7 days after the initial surgery.

## 4. Discussion

### 4.1. Survival

OS rates did not differ significantly in the TORS and TLM groups either 2 or 3 years after surgery (*p* = 0.42; *p* = 0.20). LRFS, RRFS, and MRFS were higher at 2 and 3 years in the TORS group, but without a significant difference. Previous studies comparing the results of TLM and TORS for all OPSCC sites have not revealed any differences in OS or recurrence-free survival (RFS) [15,17]. However, these studies are retrospective and do not systematically report HPV status, tobacco and alcohol history, or tumor size. In a retrospective study of 130 patients with T1-T2 OPSCC managed by TORS or open approaches, Ford et al. [19] found that RFS at 3 years was 89% after TORS versus 73% after open surgery (*p* = 0.035). More specifically, for BOT SCC, Chillakuru et al. [16] found that OS was higher in both HPV-positive and HPV-negative patients treated by TORS than in those treated with TLM or open surgery. 

### 4.2. Margins

Similar rates of histological margin invasion were observed in the two groups (*p*= 0.45). These results are consistent with a registry study of over 2000 patients treated by TORS, over 300 TLM patients, and over 6000 patients treated by other surgical techniques for T1-2 OPSCC, which showed significantly more positive margins for all non-robotic surgical techniques (*p* < 0.001) except for TLM (*p* = 0.582) [17]. Regardless of the technique chosen for transoral surgery, two limitations must be considered in the analysis of margins. Specimens may be fragmented because of piecemeal resection, compromising histological margin analysis, but oncological results remain good [20], especially if frozen sections are used, as shown in the meta-analysis of Gorphe et al. [13]. Still, most of the time, en bloc resection is possible in BOT surgery; in our study, all resections were macroscopic en bloc resections. To account for the effects of coagulation shrinkage, accessibility, and function preservation, the threshold for clear margins might be set at 2, 3, or 5 mm depending on local practices in the different pathology departments [21]. 

### 4.3. Tumor Size

Tumors removed by TORS were significantly larger than those removed by TLM (*p* = 0.04), despite no significant difference in the staging distribution of the tumors. This has already been reported previously [22] and can be explained by the better 3D visualization of the operation field in TORS and by the simultaneous use of several instruments in a comfortable position by the surgeon. This highlights the limits of the TNM classification, as it is often easier for the surgeon to remove a large but exophytic tumor than a small endophytic one [23].

### 4.4. Functional Respiratory and Feeding Follow-Up

Direct access to the tumor in transoral approaches reduces postoperative functional morbidity compared with traditional open approaches, mainly for swallowing [24]. Initial enteral feeding was far from systematic, with nasogastric feeding tubes for 50% of patients in the TLM group and in 86% of those in the TORS group and for short durations (median < 8 days). The importance of the BOT for swallowing explains why more patients in our cohort received enteral feeding than in Sumer et al.’s (38% in the TLM group and 24% in the TORS group) which included tumors in all OPSCC sites [12]. Long-term gastrostomy feeding need did not differ between the two groups (12% and 13%). All patients with long-term gastrostomy had a history of prior cervical radiation or postoperative radiation which may impair swallowing function. Moreover, none of our patients had flap reconstruction that could avoid secondary healing contractions and functional disorders.

Tracheotomy was performed for less than one in five patients in the TORS group but none at all in the TLM group (*p* = 0.09). The procedure was performed prophylactically to limit the risk of surgical site bleeding or edema, particularly in large resections, with relatively rapid cannula removal (10 days on average). The absence of secondary tracheotomies suggests that they are not essential; TLM was only performed in one of the six study centers, so the difference with the TORS group most likely highlights local guidelines on the use of tracheotomy. The proportion of patients who underwent primary tracheotomy in the TORS group is consistent with those reported in the literature [8,25,26,27].

### 4.5. Operating Time/Hospitalization

Operating times were 16 min (roughly 20%) shorter in the TLM group (*p* = 0.03). The clinical relevance of this reduction in operating time is debatable relative to oncological and functional criteria and given the size of the lesions encountered. As TORS-removed tumors were significantly larger than the TLM-removed, ones they might have been more delicate and thus require more time to remove. Postoperative lengths of stay were not significantly different, but a difference of 3 days in median lengths of stay (longer after TORS) may be clinically relevant for ward turnover. This might be due to a more frequent use of preemptive tracheostomy. The result for the operating times is contrary to that of Sumer et al. [12], who found that TORS procedures were shorter than TLM on average, but in keeping with the results of Weinstein et al. [28], who found, considering all tumor locations, that operating times with “classic” transoral approaches such as TLM were on average 4 min shorter than those with TORS. The time required to set up the robot decreases with the surgical team’s level of experience, and once in place, the robot requires fewer adjustments than may be required in TLM to successively change and adjust laryngoscopes. Sievert et al. [15], with a larger study population, found that neither technique was superior in terms of operative times or hospital lengths of stay. 

### 4.6. Safety: Postoperative Bleeding

Around 13% of patients in the TLM and the TORS group had postoperative bleeding. Pollei et al. [22] likewise found no significant difference between TLM and TORS groups in an analysis of 906 resections of all sites of OPSCC (5.6% in the TLM group and 5.9% in the TORS group; *p* = 0.80). The higher rate in our series can be explained by the BOT being more prone to bleeding. The risk factors for bleeding reported in the literature are male gender, high T stage, and high blood pressure [22,29]; our study group was too small (54 patients) to analyze risk factors, but it is noteworthy that among the eight patients who had bleeding complications, four were male, three had a T2 SCC, and three had a known history of high blood pressure.

### 4.7. Strengths

This study’s strengths include the inclusion of patients from six French tertiary reference hospitals. It is representative of patients managed in routine practice, with a large proportion of recurrences and second cancers that might not be eligible for a chemo-radiation treatment. It shows satisfactory oncological control, functional results, and safety in these patients. It is the first study to focus specifically on the BOT, which is often a problematic site, and supports the conclusions of previous studies comparing the effectiveness and safety of robotic surgery in the oropharynx. On this basis, we recommend widening the use of robot-assisted surgery, to improve patient outcomes, increase surgeon comfort, and decrease TORS costs.

### 4.8. Limitations

The main limitation of the study is its small population, which precluded any multivariate statistical analysis of known prognostic factors for OPSCC such as HPV status, TNM stage, and previous radiotherapy. Follow-up in the TLM group was shorter: 14.4 months against 37.2 months for TORS; this might be why we could not reach statistically significant differences between the groups in survival analysis. Nevertheless, the preliminary descriptive analysis of the two groups, which revealed no significant differences in these variables between groups, suggests no substantial bias was induced. The study was also limited by missing data, due to its retrospective design, and losses to follow-up.

## 5. Conclusions

Both TORS and TLM showed encouraging oncological, functional, and safety results in BOT SCC even in recurrence or second primary cancer patients, without a technique being found superior in terms of OS or RFS. Tumors removed by TORS were larger without an increase in postoperative bleeding. The shorter operating times and lower costs observed for TLM seem clinically less relevant factors in choosing between the two techniques. This study suggests that TORS is a useful tool for extending the possibilities of transoral treatment maybe even to more extended BOT SCC with the possibility of combination with reconstructive technics.

## Figures and Tables

**Table 1:** Patient preoperative characteristics.

	TLM *n* = 16	TORS *n* = 38
Age (years)	66 (±7)	63 (±8)
Male gender	10 (63)	32 (84)
History of UAT SCC *	10 (62.5)	16 (42.1)
- with radiotherapy treatment history	9 (90)	12 (60)
- time between last UAT SCC * and BOT SCC ** (months)	18 [12.5–50.3]	19 [1–64]
Preoperative ASA score	(*n* = 16)	(*n* = 37)
- 1	- 1 (6)	- 14 (37.8)
- 2	- 11 (68.8)	- 19 (51.4)
- 3	- 4 (25)	- 4 (10.8)
cT stage		
- 1	- 8 (50)	- 21 (55.2)
- 2	- 8 (50)	- 17 (44.8)
cN stage		
- 0	- 9 (56.2)	- 15 (40.6)
- 1	- 5 (31.2)	- 12 (32.4)
- 2	- 2 (12.5)	- 10 (27)
Lymph node dissection	7 (43.8)	26 (68.4)
Intraoperative complementary sections	13 (81.3)	37 (82.2)
Lingual artery ligature	(*n* = 16) 2 (12.5)	(*n* = 34) 10 (27)
Follow-up (months)	14.4 [7.2–27.6]	37.2 [13.2–64.8]

Results as number (percentage) for categorical variables, mean (standard deviation) for age, and median [interquartile range] for continuous time variables. * UAT SCC: upper aerodigestive tract squamous cell carcinoma; ** BOT SCC: base of the tongue squamous cell carcinoma.

**Table 2:** Tumor characteristics.

	TLM *n* = 16	TORS *n* = 38	*p*-Value
Positive margins	4 (25)	6 (15.7)	0.45
Tumor size (mm)	31 (±10)	39 (±14)	0.04
pT stage			0.35
- 0	- 1 (6.2)	- 0 (0)	
- 1	- 10 (62.5)	- 18 (47.4)	
- 2	- 5 (31.2)	- 20 (52.6)	
pN stage			0.39
- 0	- 1 (6.2)	- 6 (15.8)	
- 1	- 4 (25)	- 9 (23.7)	
- 2	- 2 (12.5)	- 11 (28.9)	
- X	- 9 (56.3)	- 12 (31.6)	
HPV * positive	(*n* = 15) 8 (53.3)	(*n* = 29) 13 (44.8)	0.58

Results are expressed as number (percentage) for categorical variables and mean (standard deviation) for continuous variables. * HPV, human papilloma virus.

**Table 3:** Functional results.

	TLM (*n* = 16)	TORS (*n* = 38)	*p*-Value
Operating time (min)	59 [24–96]	75 [55–158]	0.03
Hospital stay (days)	5 [2.5–9]	8 [7–11]	0.2
Tracheotomy	0 (0)	7 (18.4)	0.09
Nasogastric feeding tube - Duration (days)	8 (50) 4 [2.8–9.8]	26 (86.4) 7 [5.8–34.2]	0.33 0.61
Gastrostomy	2 (12.5)	5 (13.2)	1

Results are expressed as number (percentage) for categorical variables and mean (standard deviation) for continuous variables.

## Data Availability

The data presented in this study are available on request from the corresponding author. The data are not publicly available due to ethical restrictions.
